# Suspension height tune-up with constant stiffness properties through motor-driving double-gas-chamber hydro-pneumatic strut: Experimental study and modeling

**DOI:** 10.1371/journal.pone.0314529

**Published:** 2024-11-26

**Authors:** Ruihong Li, Fan Yang, Feng Zhao, Weiqiang Zhang, Dezhao Lin

**Affiliations:** 1 Research Center for Intelligent Materials and Structures (CIMS), College of Mechanical Engineering and Automation, Huaqiao University, Xiamen, Fujian, P. R. China; 2 College of Marine Engineering, Jimei University, Xiamen, Fujian, P. R. China; 3 Xiamen TVS Test Science and Technology Co., Ltd, Xiamen, China; Federal University of Technology - Parana, BRAZIL

## Abstract

In vehicle suspension, it is important to achieve continuous height adjustment to reduce the possibility of unstable and off-tracking caused by uneven postures. It is usually solved by air suspension, in which the dynamic properties will change under the adjusting process and these changes are not conducive to control. Considering the above, in this paper a double-gas-chamber hydro-pneumatic strut (DHPS) with the constant and/or predicted stiffness during continuous height adjustment, as masses (oil and gas) conversation are guaranteed in the whole system, which is achieved by volume variation of auxiliary gas chamber through motor-driving piston, is proposed. The dynamic properties and mathematic model of the proposed DHPS are investigated and established through bench test applying to the designed prototype. The system response speed has been evaluated through experimental data that for harmonic test the system can reach the stable condition in 1 (2) cycle subjected to 50mm/s (25mm/s) motor-driving piston moving speed. Finally, a typical quarter-car model is utilized to evaluate the performance of the proposed DHPS. It has been shown that the system takes 0.40s and 0.50s (200mm/s moving speed), and 0.76s and 1.36s (40mm/s moving speed) subjected to step test (25 and 50 mm), respectively.

## 1. Introduction

The vehicle suspension mainly provides the function of supporting, guiding, vibration suppression, and stability of vehicles [1]. Except for the above, the vehicle suspension system plays an important role in vehicle attitude (leveling) control, which is mainly decided by the extension of each suspension strut [2]. The traditional passive suspension will be tuned to maintain the leveling of vehicle posture subjected to the designed loading condition/range. The distribution of sprung mass, which is also associated with the number, weight, and seat distribution of the vehicle passengers or loads, cannot always be stable under practical working conditions, which increases the probability of rollover and affects security [3]. Secondly, it is well known that effective posture control through suspension systems subjected to different distributions of the sprung mass and road conditions can greatly improve ride comfort performance [4]. To meet the increasing requirement of ride comfort and handling performance, it has become one of the research hotspots to modify the suspension height [5, 6].

Recently, to meet the requirement of vehicle height adjustment under different driving states, some structures designed based on air and/or hydro-pneumatic suspensions are proposed to achieve height adjustment function during driving [7, 8]. In this kind of structure, the pumps and the solenoid valves are normally required to achieve the function of inflation and deflation procedures [9], which is normally associated with high impact and then affecting the ride comfort and handling performance [10]. And larger space is normally required to contain the complex system including the hydraulic oil/gas pump, oil/gas tank, hydraulic pipe, and other components [2]. Inflation and deflation procedures with high frequency will significantly increase the working temperature, especially the pumping system [11]. Besides, the low response speed is expected as the selected pumping working method. Here, it should be emphasized that as the extra mass of air and/or hydraulic oil pumping input/output the system, the total volume (gas or hydraulic oil) of the whole system cannot be constant, and then the system’s dynamic properties (especially stiffness property) will change during height adjustment procedure [12]. It is difficult to predict the system’s dynamic properties during inflation and deflation procedures. It is hard to distinguish the advantages or disadvantages of the variation of the stiffness property with the height adjustment, as it depends on different working conditions. However, it may be a better choice for the suspension system to conduct the independent height adjustment with constant and/or predicted suspension stiffness properties, which may be convenient for the related controller design.

Based on the above, one methodology of height adjustment with constant (predicted) stiffness property will be proposed based on the compacted double-gas-chamber hydro-pneumatic strut (DHPS) [13]. The volume of the extra gas chamber in the DHPS will be modified through a motor-driving piston. As no extra mass of air in/out of the system, the stiffness property of the strut will be constant and/or predicted during the height adjustment procedure. Compared with the traditional pumping method, high response speed can be achieved as the selected motor driving method. The working principle of the proposed motor-driving DHPS and the experimental study applying it to the designed prototype will be presented in Section 2. In Section 3, the mathematical model of the proposed motor-driving DHPS will be established based on the experimental study. In Section 4, the validity of the established model will be verified through the comparison between the experimental data and simulation results, in which the coefficient of determination (*R*^*2*^) are all above 0.92. And then the validity of the proposed DHPS will be verified through a typical quarter-car model subjected to different excitation signals and sprung masses. Here, It should be noted that the proposed DHPS can be also utilized in other multi-point support structures in different fields, such as ocean platforms, engineering machinery, platform tensioner-riser coupling systems, and so on [14, 15].

## 2. Working principle and experiment study

In this section, the working principle associated with the proposed motor-driving DHPS will be introduced, and its dynamic properties will be evaluated through the established test bench.

### 2.1 The structure and working principle

**Fig 1:**
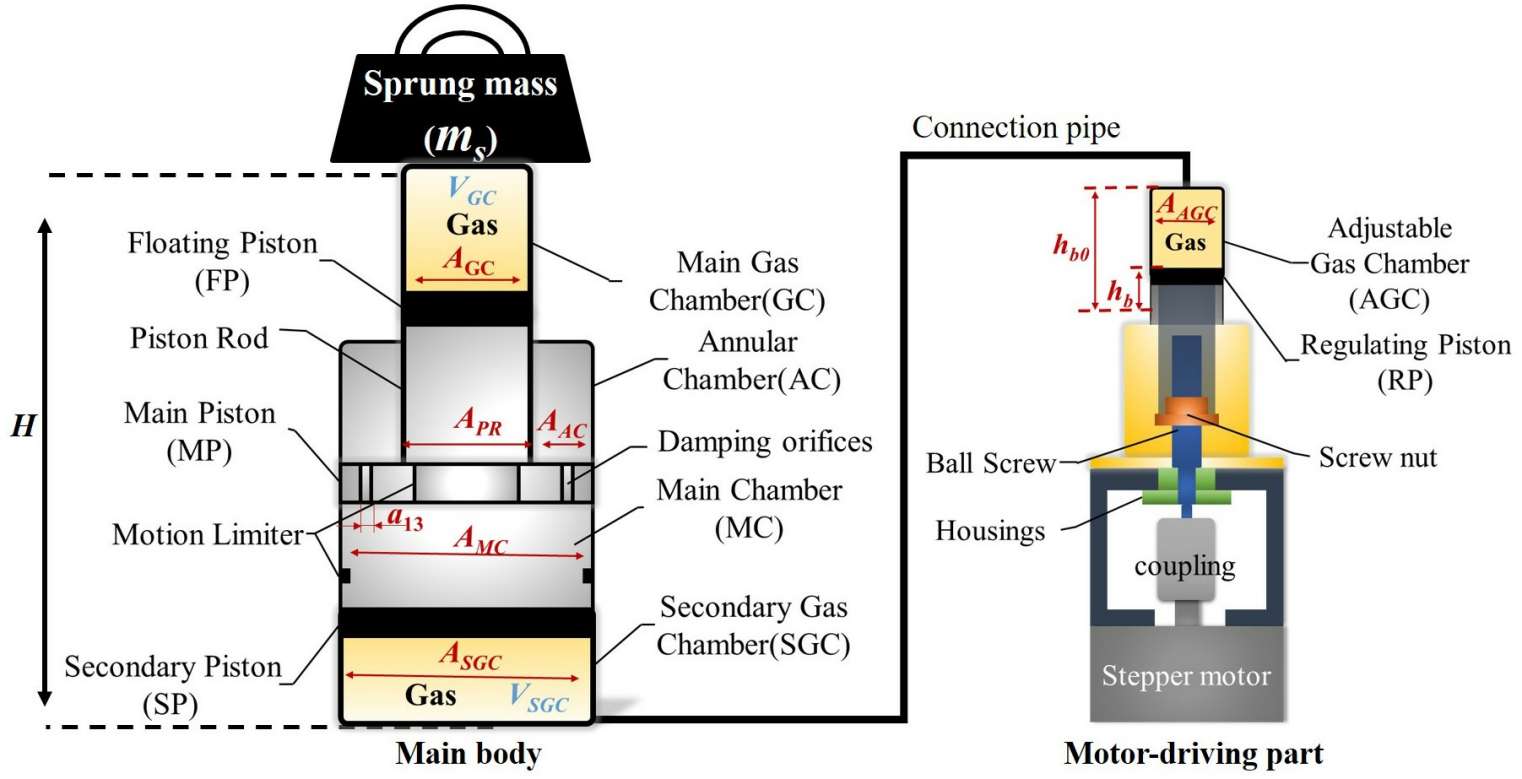
The schematic of the proposed motor-driving DHPS.

A schematic of the motor-driving DHPS, as shown in Fig 1, is composed of two parts: main body (DHPS) and motor-driving part. DHPS consists of two compacted gas chambers, which are Main Gas Chamber (GC) and Secondary Gas Chamber (SGC). There is an Adjustable Gas Chamber (AGC) in motor-driving part. The SGC and AGC are connected by a connection pipe, and the volume change of AGC by Regulating Piston (RP) will not affect the total volume of AGC and SGC under the same sprung mass. Damping orifices on the Main Piston (MP) allow the flow between the Annular Chamber (AC) and Main Chamber (MC). Floating Piston (FP) and Secondary Piston (SP) are utilized to separate the oil and gas to avoid emulsification [16]. The physical parameters of the design prototype shown in Fig 1, are summarized in Table 1. Here, the parameters of the quarter-car model have also been summarized in Table 1.

**Table 1:** The physical and initial parameters of the motor-driving DHPS prototype and quarter-car model.

Definition	Description	Value
*A*_*1*_	Area of MC	3117.20mm^2^
*A*_*2*_	Area of the piston rod	1590.40mm^2^
*A*_*3*_	Area of AC	1526.80mm^2^
*A*_*GC*_	Area of GC	883.50mm^2^
*A*_*SGC*_	Area of SGC	3117.20mm^2^
*A*_*AGC*_	Area of AGC	1256.60mm^2^
*ρ*	Density of the oil	797.00kg/m^3^
*C*_*d*_	Flow coefficient	0.70
*n*_13_	Number of orifices between MC and AC	2.00
*a*_13_	Area of orifice	8.04mm^2^
*V*_*GC*0_	Maximum volume of GC	224.00cm^3^
*V*_*SGC*0_	The sum of the maximum volume of SGC and AGC	385.00cm^3^
*h*_*b*0_	Maximum length of AGC	104.00mm
*P*_*GC*0_	Pre-charge pressure in the GC	0.70MPa
*P*_*SGC*0_	Pre-charge pressure in the SGC	0.70MPa
*D*_*p*_	Diameter of connection pipe	8.00mm
*L*_*p*_	Length of connection pipe	700.00mm
*k*_*t*_	tire stiffness [17]	190.00kN/m
*m*_*s*_	sprung mass [17]	320.00kg
*m*_*t*_	unsprung mass [17]	45.00kg

**Fig 2:**
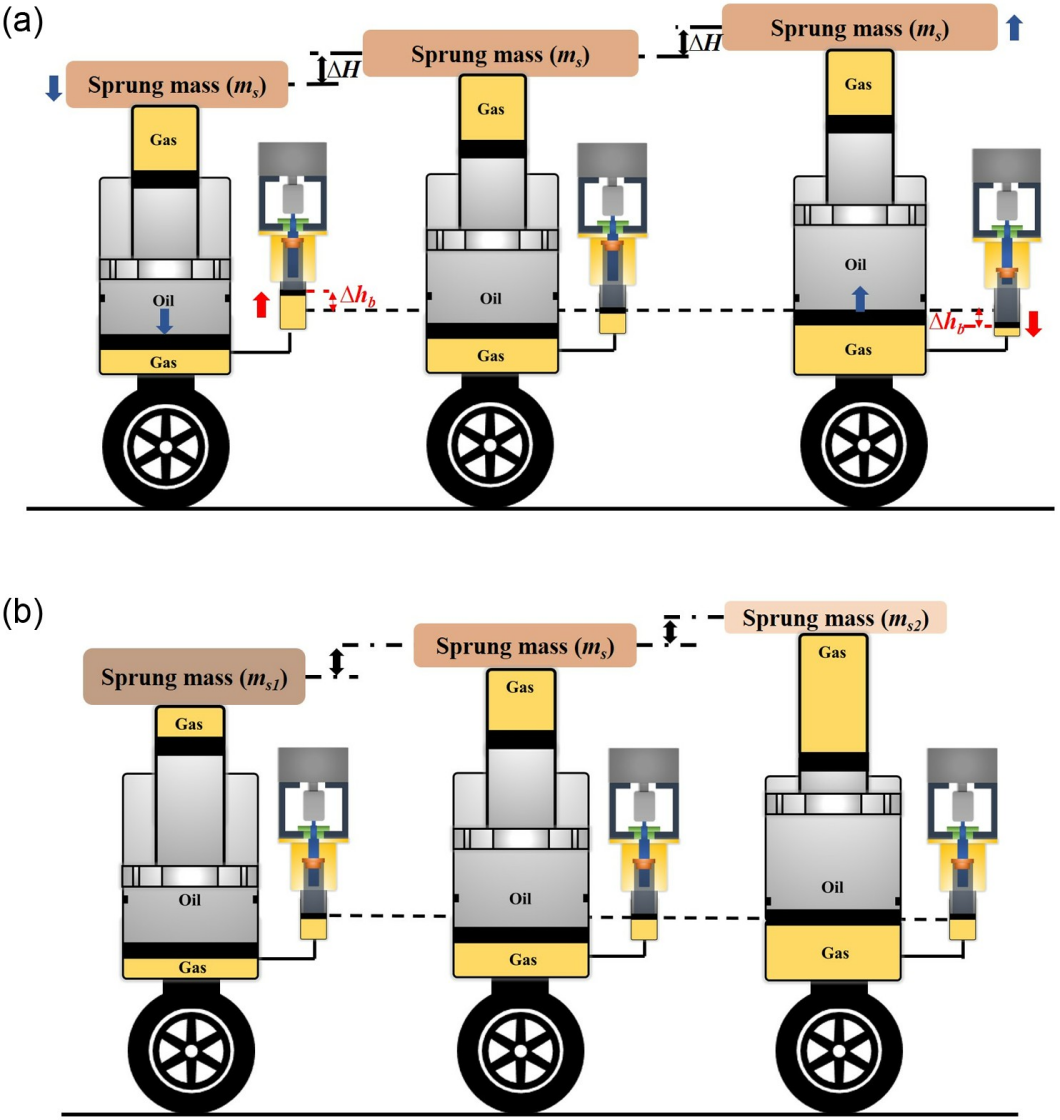
The working principle of the motor-driving DHPS. (a) The height adjustment process (b) The strut height under different sprung masses (*m*_*s2*_*<m*_*s*_*<m*_*s1*_).

The output force of DHPS under a static state (*P*_1_ *= P*_3_ = *P*_*static*_) ignoring friction forces can be expressed as [13]:

$$F=P_{1}\cdot A_{1}-P_{3}\cdot A_{3}=P_{static}\cdot A_{2}$$ (1)

where *P*_1_, *P*_3_, and *P*_*static*_ represent pressures of MC, AC, and static state, respectively, which are all equal under the static state, and then

$$P_{static}=F/A_{2}=m_{s}g\;/A_{2}$$ (2)

where *m*_*s*_ and *g* represent the sprung mass and gravitational acceleration (9.8N/kg). The relationship between pressure and volume under the ideal gas law can be expressed as:

$$PV^{n}=Constant$$ (3)

where *P* represents the absolute gas pressure (*P*_*static*_ + atmospheric pressure) under static state; *V* represents the gas volume; *n* represents the polytropic exponent. Substituting Eqs (2) and (3), one can obtain:

$$V_{SGC}=\sqrt[{n}]{\left(Constant\times A_{2})/(m_{s}g\right)}$$ (4)

where *V*_*SGC*_ represents the volume of SGC and AGC. Based on Eq (4), one can utilize the volume of AGC, which is associated with the value of *h*_*b*_, to modify the volume of chamber SGC to obtain the required strut height *H* shown in Fig 1 as:

$$\Delta H=\Delta V_{SGC}/(A_{1}-A_{3})=\Delta V_{AGC}/A_{2}=A_{AGC}/A_{2}\cdot \Delta h_{b}$$ (5)

where *ΔV*_*SGC*_ and *ΔV*_*AGC*_ represent the variation volume of SGC and AGC, respectively; *A*_*AGC*_ represents the area of AGC; *Δh*_*b*_ and *ΔH* represent the variation of the motor-driving piston and strut height, as shown in Fig 1. Fig 2 illustrates the fundamental working procedure of the strut height adjustment (*H*) subjected to the same and different sprung masses.

**Fig 3:**
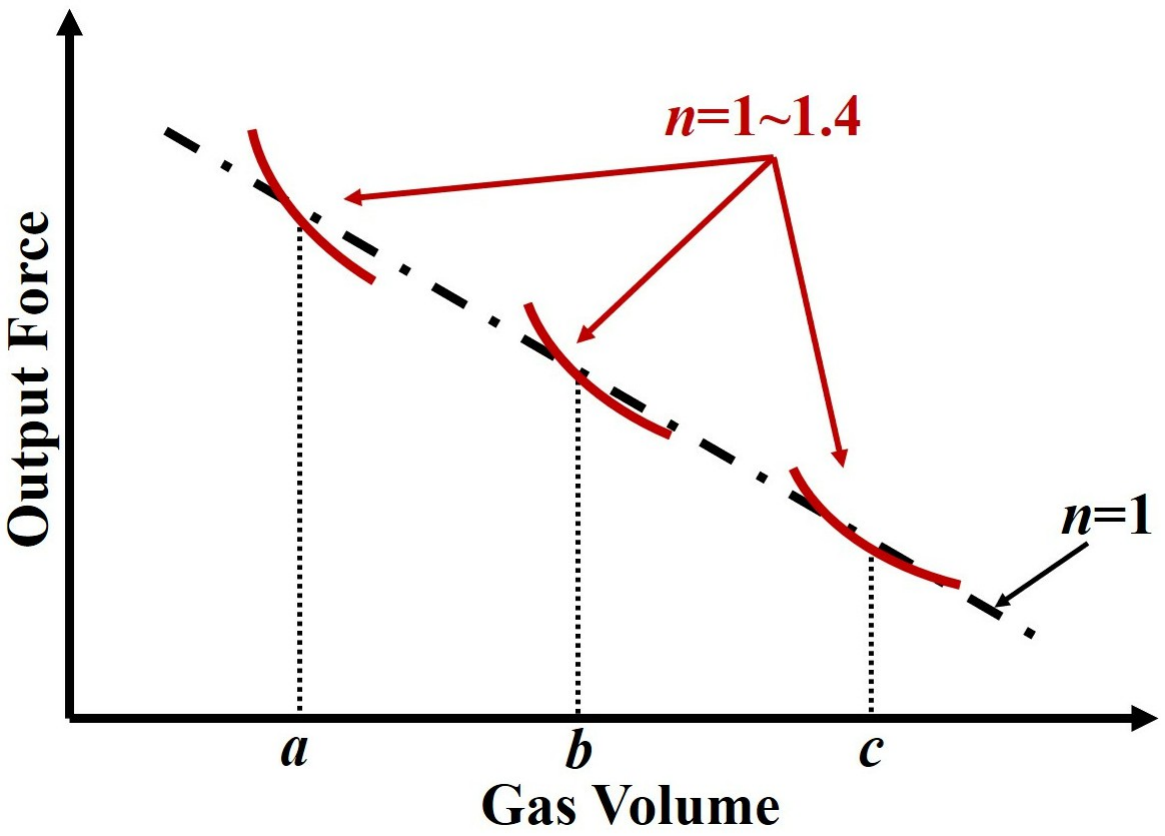
The output force curve diagram of motor-driving DHPS. Dashed line (black): static output curve; Solid line (red): working dynamic curve.

According to the working principle of the hydro-pneumatic strut, the gas variation is closer to the isothermal process under the static state, in which the polytropic exponent (*n*) is equal to 1. Based on the above analysis, one can find that the static stiffness property (*n* = 1) of the system under different sprung masses. This property is expressed by the slope of output force, which consistently follows the curve shown in Fig 3 (dashed-dotted line for *n* = 1, according to Eqs (1) and (2)). During working conditions with a specific sprung mass, the system stiffness will follow Eq (3) subjected to different *n* values (normally from 1 to 1.4 [18]), as shown in Fig 3 (solid red line, the *a*, *b*, *c* represent gas volume under different sprung masses). Therefore, one can find that the stiffness property is constant and/or predicted in the height adjustment process, which is different from other height adjustment methods. This kind of height adjustment method with constant and/or predicted stiffness properties will be convenient for the control system design of active suspension design.

### 2.2 Experiment study

**Fig 4:**
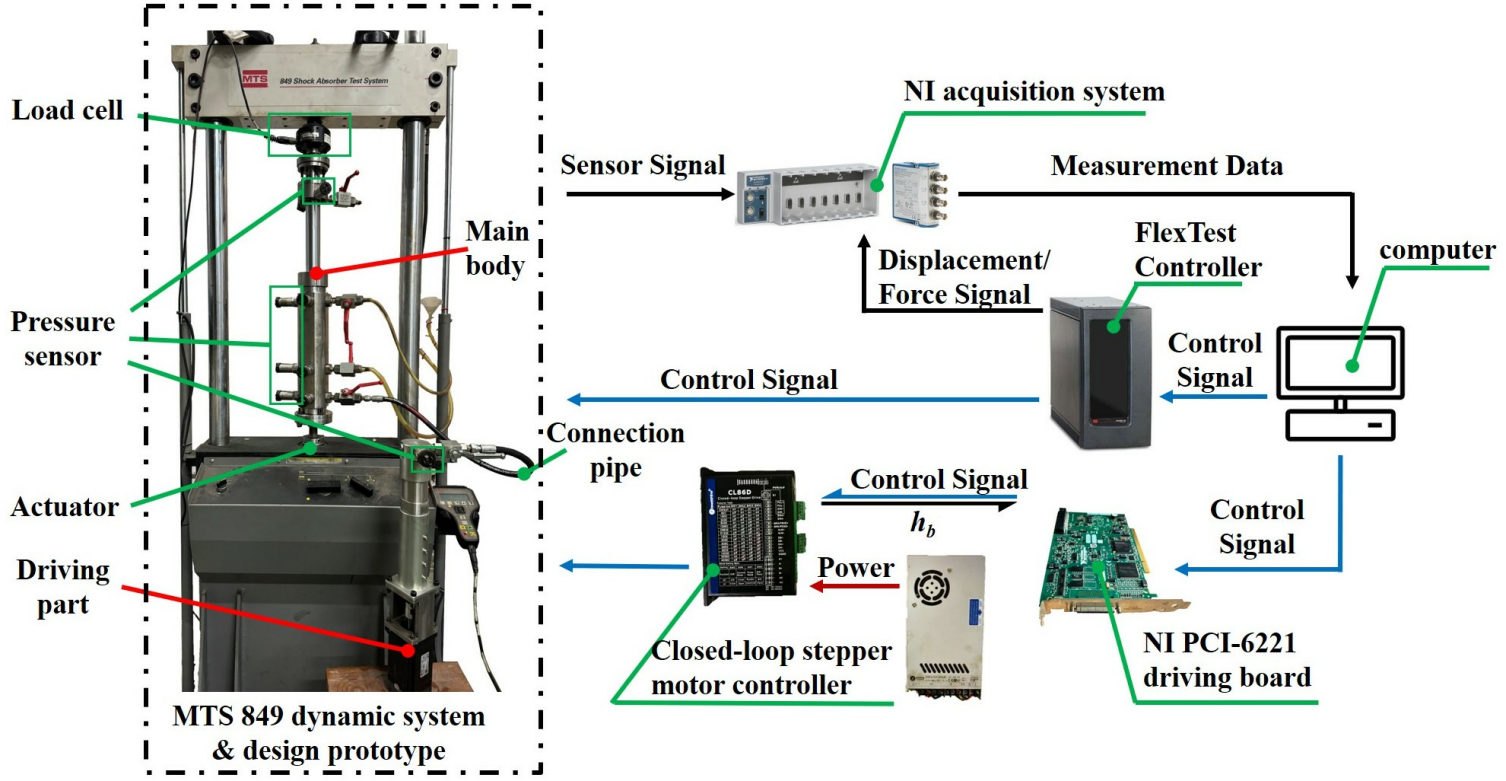
The experimental setup.

The test bench of Dynamic Mechanical Analysis (DMA) shown in Fig 4, which consists of the MTS 849 dynamic system (with an embedded load cell (0-10kN) and an embedded linear variable differential transformer (LVDT, 0-250mm)), closed-loop stepper motor controller, NI acquisition system, has been established to evaluate the dynamic properties. The RP is driven by a closed-loop stepper motor and controlled through the NI PCI-6221 driving board with customer software. Several pressure sensors (0-10MPa) are installed to measure the gas/oil pressure. At least 30 cycles have been measured for each excitation to ensure the stability of output characteristics.

Here, it should be noted that the designed DMA test bench cannot directly evaluate the dynamic properties of the proposed DHPS subjected to different values of *h*_*b*_ and sprung masses under base excitation, as shown in Figs 2(b) and 3. However, according to the working principle of DHPS, the different sprung masses in Fig 2(b) will lead to the different initial value of the load cell, which can be equivalent to the different value of *h*_*b*_ under DMA test, as shown in Fig 4.

Considering the above, three kinds of experimental studies have been conducted to illustrate the fundamental dynamic properties: **Test 1**: harmonic excitation with different amplitudes and frequencies subjected to the same value of *h*_*b*_. This test is utilized to evaluate the damping, stiffness, and friction properties of the proposed DHPS. The initial compression displacement is 60mm; **Test 2**: the dynamic properties subjected to different initial values of *h*_*b*_. This test is equivalent to the dynamic test for proposed DHPS under different sprung masses, as illustrated in Figs 2(b) and 3; **Test 3**: the effect of different driving speeds of RP (*v*_*b*_, Changing *h*_*b*_ with different driving speeds during motion). This test is to evaluate the output characteristic of the proposed DHPS.

**Fig 5:**
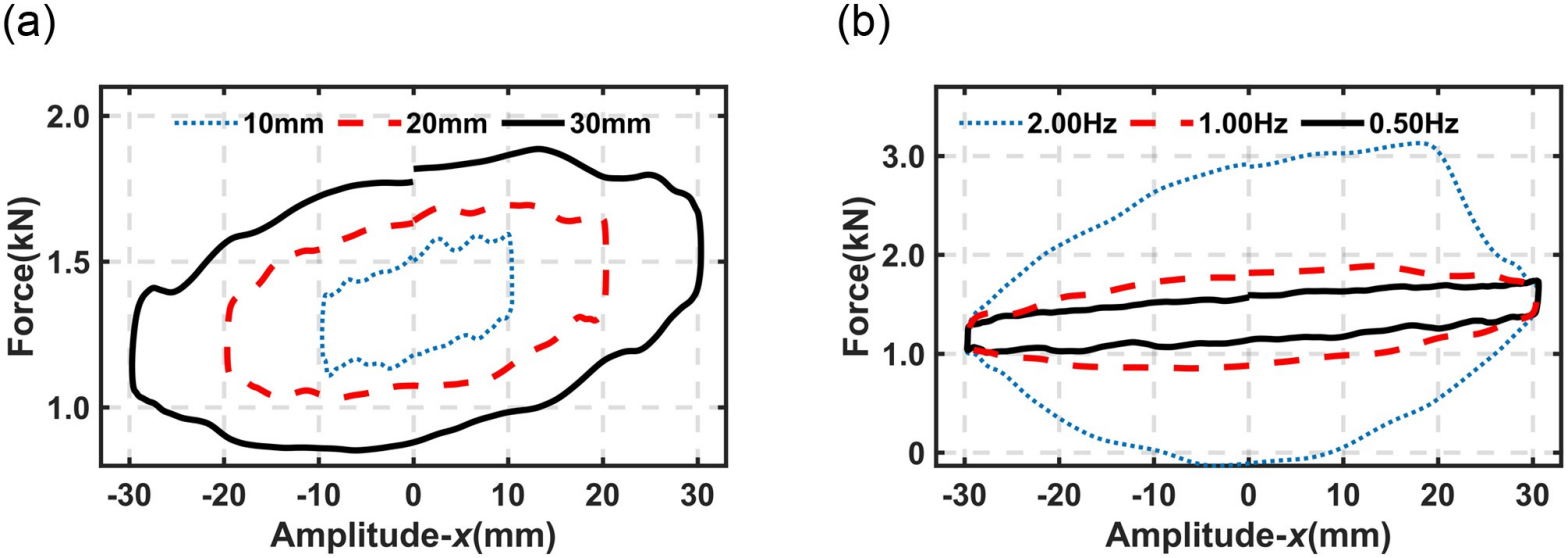
The experimental result of harmonic excitation with different amplitudes and frequencies. (a) Frequency (*f*) = 1.0Hz (b) Amplitude (*A*) = 30mm.

**Test 1.** Fig 5 illustrates the dynamic properties of the proposed DHPS subjected to harmonic excitation signals. One can find from Fig 5 that the dynamic properties, including damping, stiffness, and friction properties, are identical to the hydro-pneumatic strut system studied by previous research [13, 19]. These properties will be utilized to establish the dynamic model of the proposed DHPS, which will be presented in the next section.

**Fig 6:**
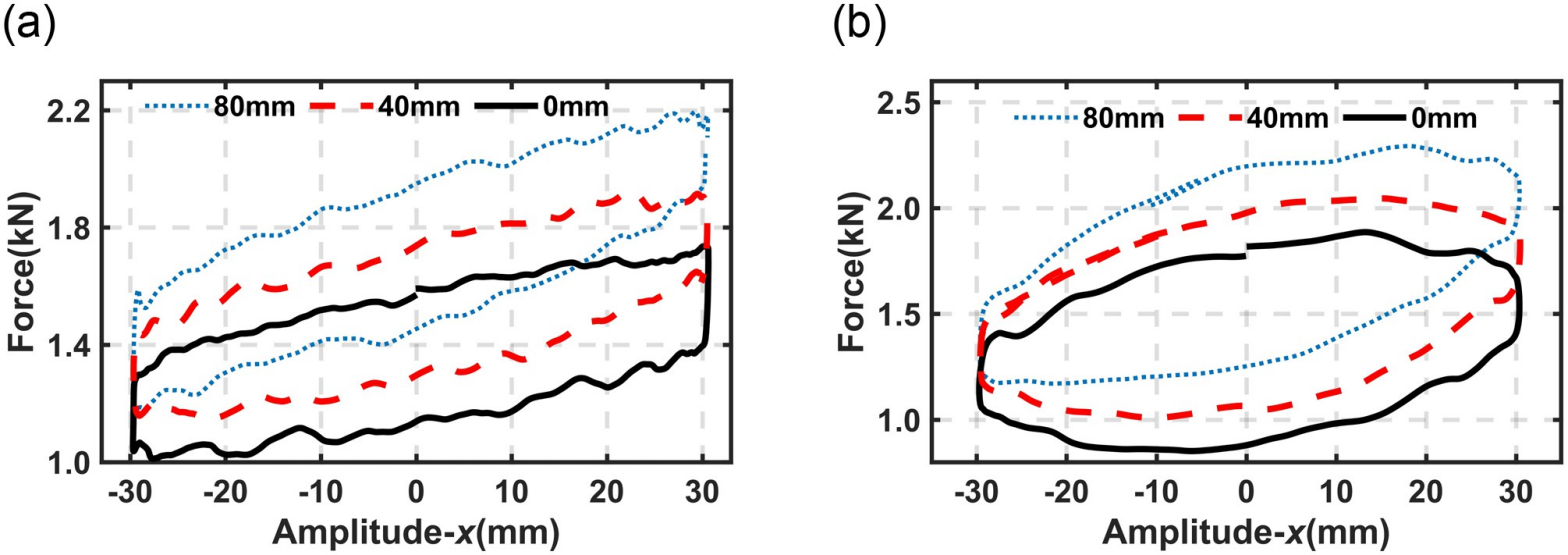
Experimental results of output force under different *h*_*b*_. (a) Frequency (*f*) = 0.5Hz & Amplitude (*A*) = 30mm (b) Frequency (*f*) = 1.0Hz& Amplitude (*A*) = 30mm.

**Test 2.** Fig 6 shows the experimental results for the proposed DHPS subjected to harmonic excitation signals under different values of *h*_*b*_. As mentioned in the previous section that this kind of test can be considered as the strut with different sprung masses through the shift of initial force, as illustrated in Fig 3. It should be noted that Test 2 is utilized to investigate the output characteristic of DHPS under different *h*_*b*_ based on DMA test (equivalent to different initial points (a, b and c in Fig 3)), in which the measured stiffness of DHPS is not constant but predicate, as different curve (red color) around points a, b and c illustrated in Fig 3. The constant stiffness is for cases with constant sprung mass subjected to base excitation under variation of *h*_*b*_.

**Fig 7:**
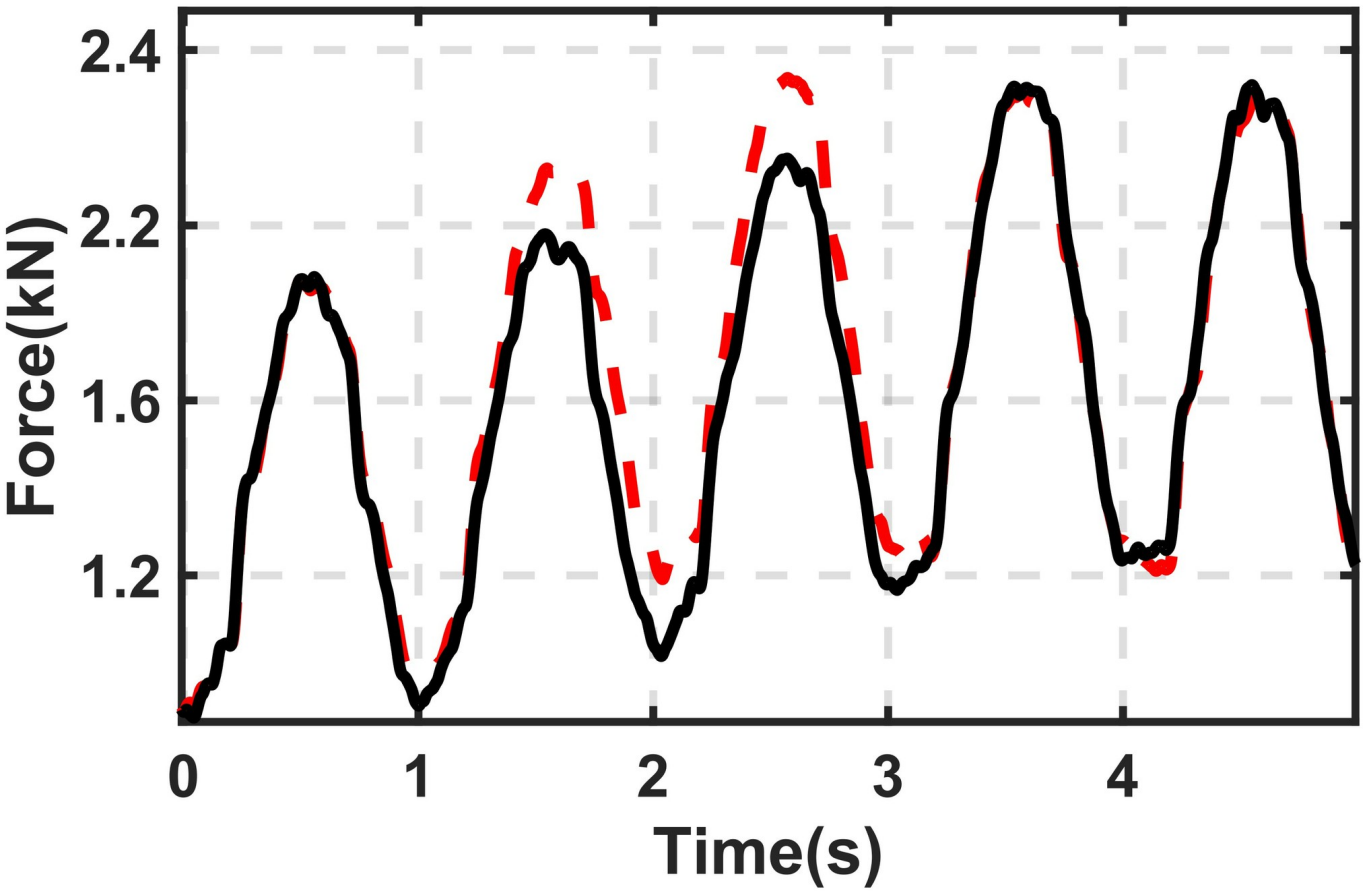
The experimental result of the *h*_*b*_ from 10 to 80mm with different driving velocities of *h*_*b*_ (*v*_*b*_ = 25mm/s & 50mm/s). Solid line (black)-*v*_*b*_ = 25mm/s; Dashed line (red)- *v*_*b*_ = 50mm/s.

**Test 3.** As presented in the introduction part, the proposed DHPS has the potential to provide good response performance compared with traditional pump-driving devices. Fig 7 shows the experimental result subjected to harmonic excitation signals with *h*_*b*_ from 10 to 80mm under different velocities (*v*_*b*_ = 25mm/s & 50mm/s). It can be found from Fig 7 that for 50mm/s, it takes less than one and a half cycles to reach the target state. In this experimental study, a simple stepper motor with a maximum torque of 8.5N∙m has been selected. To increase the response speed, one can easily select an advanced motor with high torque capacity and/or increase the area of *A*_*AGC*_.

## 3. Mathematical modelling

Based on Eq (1) and considering the effect of friction force (*F*_*f*_), the total output force of the proposed DHPS can be expressed as:

$$F=P_{1}\cdot A_{1}-P_{3}\cdot A_{3}+F_{f}\cdot \text{sign}(\dot{x})$$ (6)

where $\dot{x}$ represents the velocity of MP.

### 3.1 Elastic force

Based on the ideal gas law, the gas pressure can be expressed as:

$$(P_{i0}+P_{a})\cdot V_{i0}^{n}=\left(P_{i}+P_{a}\right)\cdot V_{i}^{n};\;\left(i=GC\;or\;SGC\right)$$ (7)

where *P*_*i*0_ and *P*_*i*_ represent the initial and working pressure of *i* chamber, respectively; *P*_*a*_ represents the atmospheric pressure (0.1MPa); *V*_*i*0_ and *V*_*i*_ represent the initial and working volume of *i* chamber, respectively; *n* represents the polytropic exponent (*n =* 1.2 [13, 19, 20]). The volume of the gas chamber can be calculated as:

$$V_{GC}=V_{GC0}-A_{GC}\cdot x_{FP}$$ (8)

where *A*_*GC*_ represents the cross-sectional area of GC; *x*_*FP*_ represents the displacement of FP. The SGC and AGC are connected by a connection pipe, and then the volume of SGC affected by the variation of the *h*_*b*_ can be expressed as:

$$V_{SGC}=V_{SGC0}-A_{SGC}\cdot x_{SP}-A_{AGC}\cdot h_{b}$$ (9)

where *A*_*SGC*_ and *A*_*AGC*_ represent the cross-sectional area of SGC and AGC, respectively; *V*_*SGC*_ and *V*_*SGC*0_ represent the sum volume of SGC and AGC, which is treated as a whole chamber in the modelling procedure, in working and initial condition, respectively; *x*_*SP*_ represents the displacement of SP, respectively. Considering the small air damping generated by the large size of the connection pipe, the effect of the resistance generated by the gas flow in the wire tube will be ignored in this study.

### 3.2 Oil damping force

The oil damping force is from damping orifices, and can be calculated through [13, 21]:

$$\left|P_{1}-P_{3}\right|=(q_{13}^{2}\cdot \rho )/[2\cdot {\left(C_{d}\cdot n_{13}\cdot a_{13}\right)}^{2}]$$ (10)

where *C*_*d*_ represents the flow coefficient (*C*_*d*_ = 0.7 [22]); *n*_13_ and *a*_13_ represent the number and cross-sectional area of damping orifices, respectively; *q*_13_ represents the flow from MC to AC which is affected by the movement of the pistons and can be expressed as:

$$q_{13}=q_{1}-q_{12}=A_{1}\cdot (\dot{x}-{\dot{x}}_{SP})-A_{GC}\cdot {\dot{x}}_{FP}$$ (11)

where *q*_1_ represents the flow out of MC; *q*_12_ represents the flow from MC to the piston rod; ${\dot{x}}_{SP}$ and ${\dot{x}}_{FP}$ represent the piston velocity of SP and FP, respectively.

### 3.3 Friction force

According to Eq (6), the friction between PR and the cylinder will directly affect the output force. The other friction force in motor-driving DHPS can be ignored. The continuous zero-velocity crossing friction model [23] is utilized to describe the friction properties as:

$$F_{f}=F_{c}\cdot c_{l}\cdot sign(\dot{x})$$ (12)

$$c_{l}=\left\{\begin{array}{cc} 0 & if\;{\dot{x}}_{i}\leq v_{0}\\ \left({\dot{x}}_{i}-v_{0}\right)/\left(v_{l}-v_{0}\right) & if\;v_{0}<{\dot{x}}_{i}\leq v_{1}\\ 1 & if\;{\dot{x}}_{i}>v_{l} \end{array}\right.$$ (13)

where *F*_*c*_ represents the normal reaction force and is set to 100 according to the experimental data; *v*_0_ and *v*_*l*_ are the given tolerances of velocities, which are set as 0 and 0.1. The gas/oil pressure in motor-driving DHPS can be expressed as:

$$P_{GC}=P_{1}=P_{SGC}=P_{AGC}$$ (14)

where *P*_*AGC*_ represents the pressure of AGC, respectively.

## 4. Discussion

In this section, the validity of the established dynamic model will be verified through experimental study. Then it will be applied to a typical quarter-car model to evaluate the fundamental performance of the proposed DHPS.

### 4.1 Model verification

**Fig 8:**
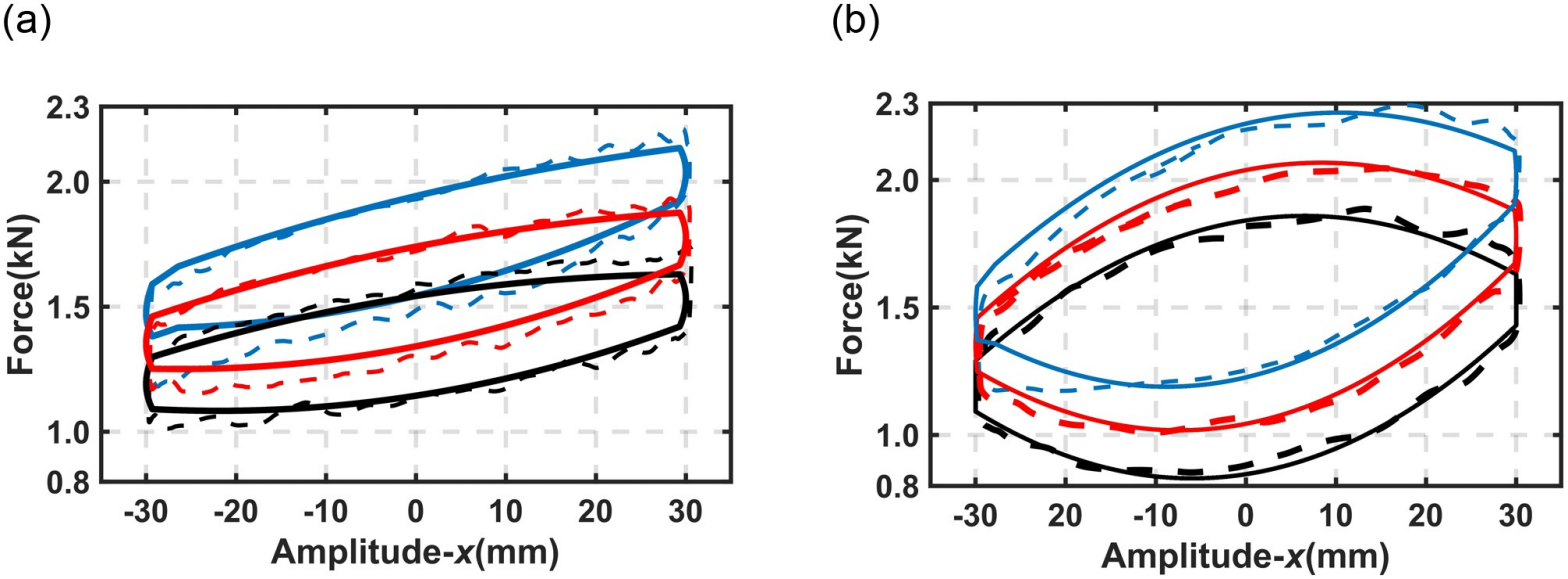
Comparison of experimental data and simulation results at 30mm amplitude under different *h*_*b*_. (a) Frequency (*f*) = 0.5Hz (b) Frequency (*f*) = 1.0Hz. Solid line-simulation result, Dashed line-experimental data; Black- *h*_*b*_ = 0mm; Red- *h*_*b*_ = 40mm; Blue- *h*_*b*_ = 80mm.

Fig 8 shows the comparison between experimental data and simulation results for the proposed DHPS subjected to harmonic excitation signals with different values of *h*_*b*_. A good match can be observed from Fig 8, and Table 2 presents the coefficient of determination (*R*^*2*^) between experimental data and simulation results.

**Table 2:** The coefficient of determination (*R*^*2*^) between experimental data and simulation result.

*f* (Hz)	*h*_*b*_ (mm)	*R* ^*2*^
0.5	0	0.93
0.5	40	0.95
0.5	80	0.92
1.0	0	0.99
1.0	40	0.98
1.0	80	0.96

**Fig 9:**
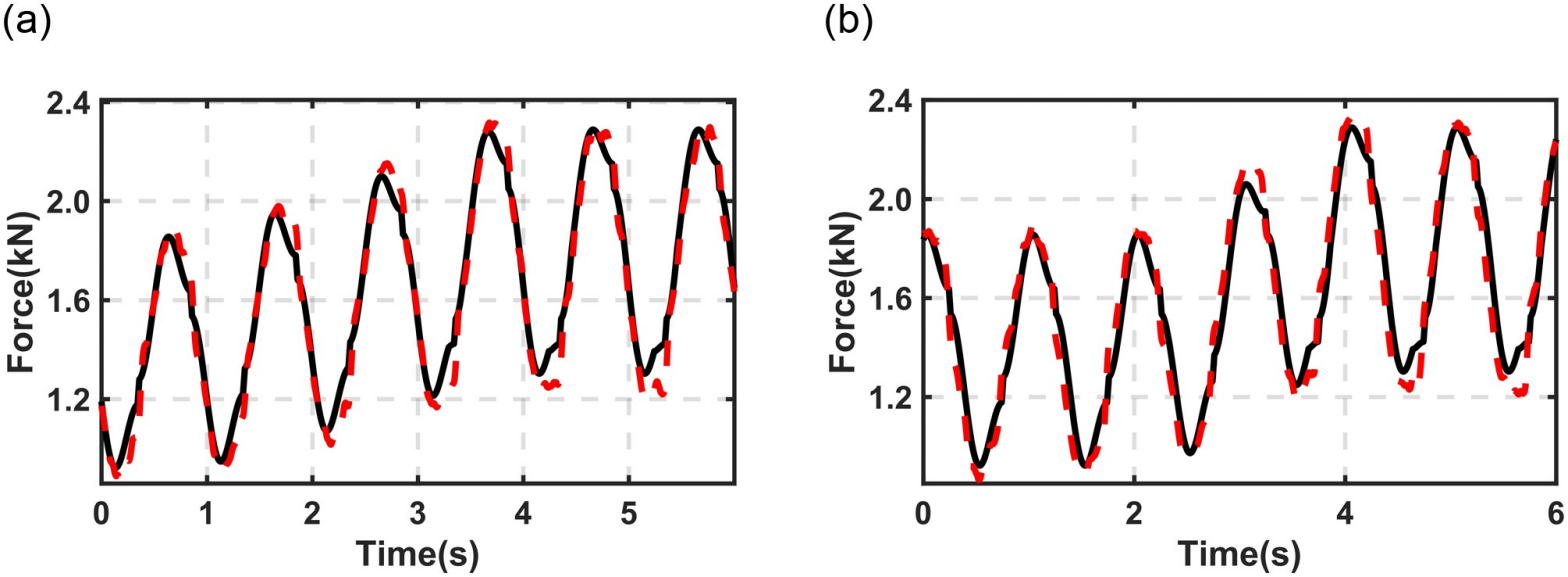
Comparison of output force between experimental data and simulation results under different *v*_*b*_ (*h*_*b*_ from 10 to 80mm). (a) *v*_*b*_ = 25mm/s (b) *v*_*b*_ = 50mm/s. Solid line (black)- experimental data; Dashed line (red)- simulation result.

Fig 9 illustrates the comparison between the simulation results and the experimental data presented in Fig 7. Again, a good match can be observed.

In the above sections, the dynamic properties, constant (predicted) stiffness properties, and response performance of the proposed DHPS have been investigated through experimental data, as illustrated in Figs 5–7. The validities of the established mathematic model of the proposed DHPS have been verified through experimental data, as shown in Figs 8 and 9. Next, the established mathematical model will be applied to a typical quarter-car model to evaluate the fundamental performance of the proposed DHPS.

### 4.2 Output characteristic under the quarter-car model

#### 4.2.1 The quarter-car model and excitation signals

The quarter-car model with the proposed motor-driving DHPS will be utilized to investigate the output performance, and the model parameters have been summarized in Table 1. The pre-charging pressure of the two gas chambers are set to 1.5MPa according to the sprung mass. The equation of motion of the quarter-car model with motor-driving DHPS can be expressed as [17, 24]:

$$m_{s}{\ddot{x}}_{s}-F\left(x_{t}-x_{s},{\dot{x}}_{t}-{\dot{x}}_{s}\right)=0$$ (15)

$$m_{t}{\ddot{x}}_{t}+F\left(x_{t}-x_{s},{\dot{x}}_{t}-{\dot{x}}_{s}\right)+k_{t}\left(x_{t}-x_{r}\right)=0$$ (16)

where ${\ddot{x}}_{s}$, ${\dot{x}}_{s}$, and *x*_*s*_ represent the acceleration, velocity, and displacement of the sprung mass relative to the ground, respectively; ${\ddot{x}}_{t}$, ${\dot{x}}_{t}$ and *x*_*t*_ represent the acceleration, velocity, and displacement of unsprung mass, respectively; *x*_*r*_ represents the input excitation. $F\left(x_{t}-x_{s},{\dot{x}}_{t}-{\dot{x}}_{s}\right)$ represents the dynamic properties of motor-driving DHPS which has been established in Section 3. A typical road excitation signal [25] will be utilized to evaluate the performance of the proposed motor-driving DHPS as:

$${\dot{x}}_{r}\left(t\right)=-2\pi f_{0}x_{r}\left(t\right)+2\pi \sqrt{G_{0}v}\cdot \;w(t)$$ (17)

where *G*_0_ is the road roughness coefficient; *v* is the vehicle speed; *f*_0_ is the lower cut-off frequency, and *w(t)* is the Gaussian white noise with zero mean value. In this study, different vehicle speeds and C-level road conditions are selected as testing signals. The *G*_0_ is equal to 256*10^−6^, and the *f*_0_ is equal to 0.011.

#### 4.2.2 System response

**Fig 10:**
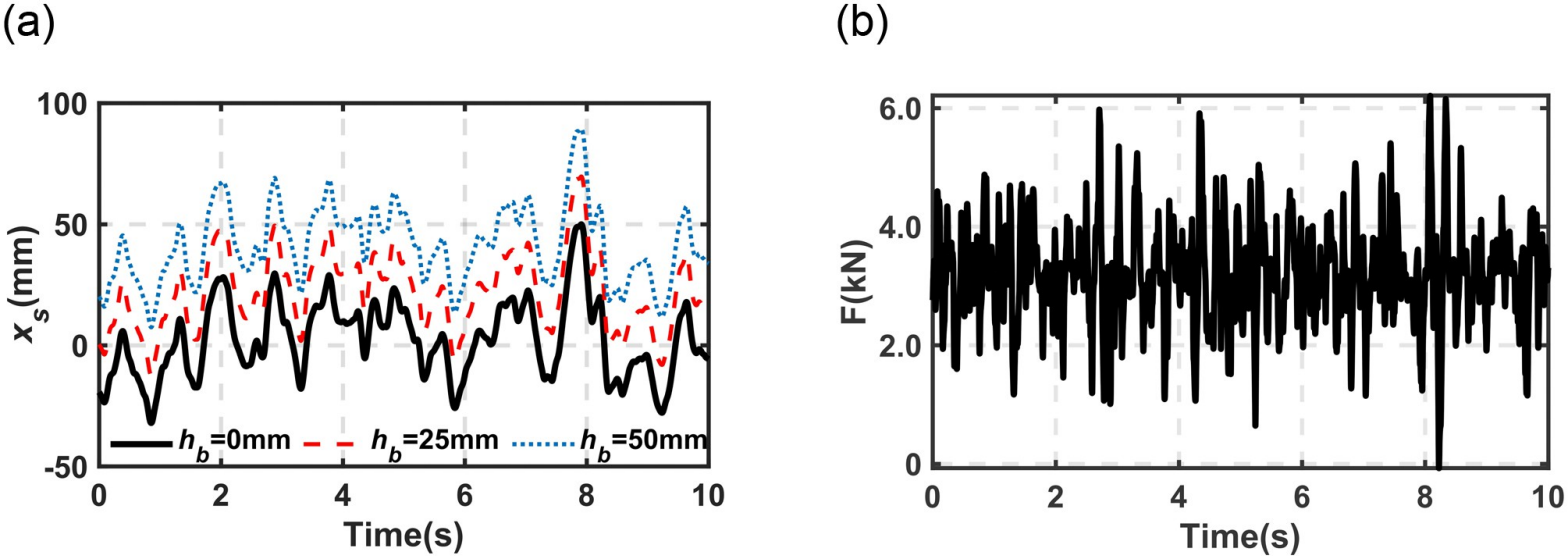
*Δ*x_*s*_ and output force with different *h*_*b*_ under the same sprung mass. (a) *Δx*_*s*_; (b) output force.

**Fig 11:**
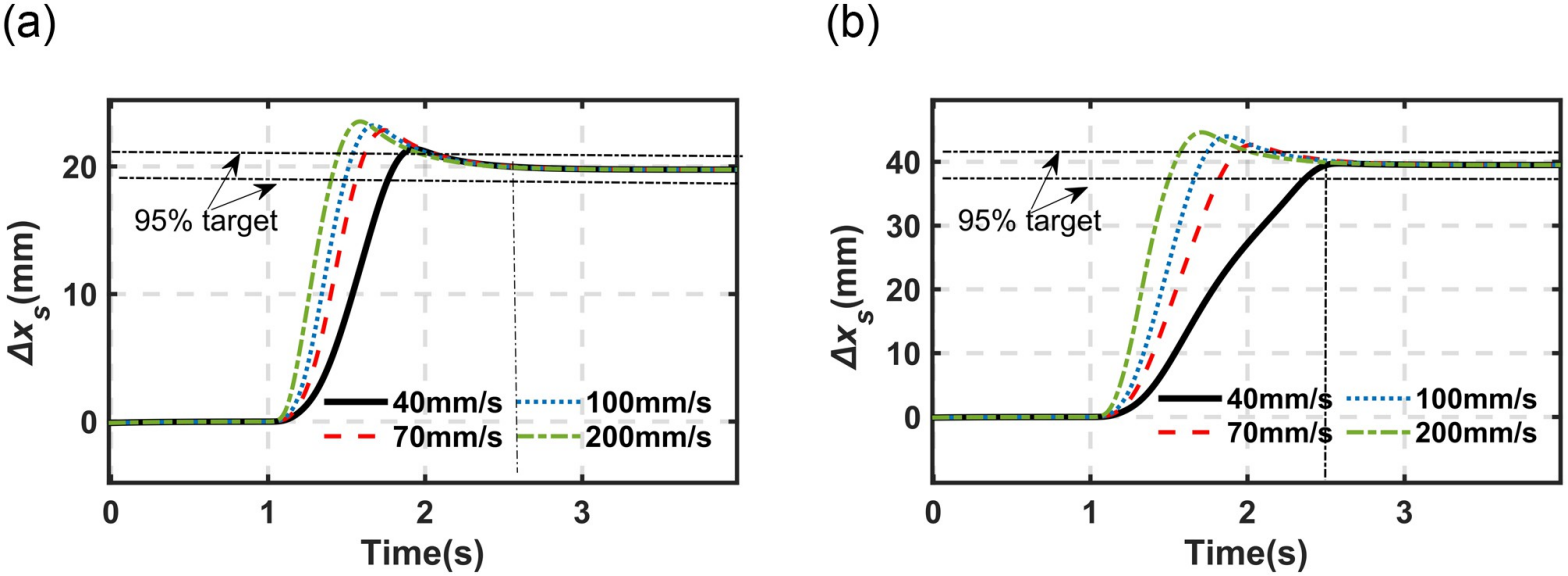
The simulation result of *Δ*x_*s*_ under a static state with different *v*_*b*_. (a) *h*_*b*_ = 0 to 25mm (b) *h*_*b*_ = 0 to 50mm.

*(1) Height adjustment subjected to the same sprung mass*. Figs 10 and 11 illustrate the response of sprung mass and the related output force subjected to different *h*_*b*_ under road excitation and harmonic excitation (Amplitude = 30mm, frequency = 1.0Hz). As discussed in Section 2, one can utilize Eq (5) to obtain the required value of *h*_*b*_. *Δx*_*s*_ represents the displacement of sprung mass (320kg) relative to the initial position of RP (*h*_*b*_ = 0mm, as shown in Fig 1). Comparing Fig 10(a), one can find that under the same sprung mass, the response of sprung mass is identical except for the shift of initial position which is decided by *h*_*b*_. Comparing Fig 10(b), the same output force from the proposed DHPS can be observed which follows the working principle of the proposed DHPS as discussed in Section 2 and shown in Fig 2.

**Fig 12:**
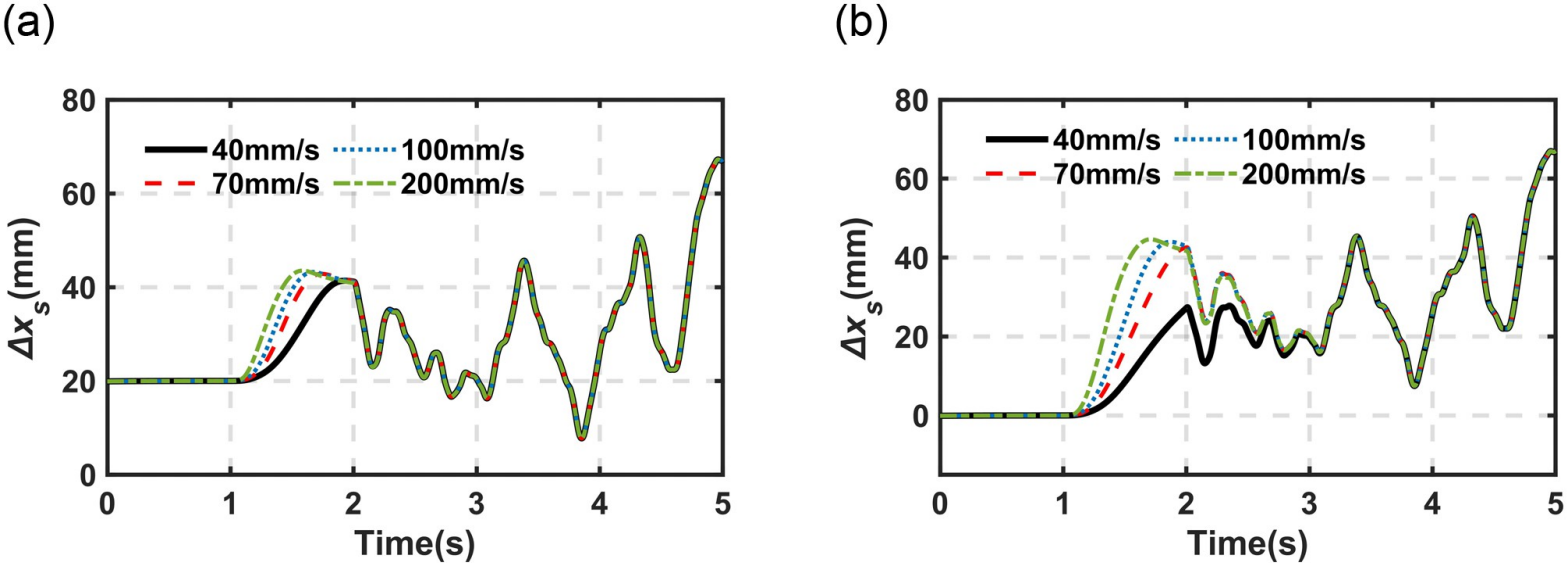
The simulation result of height adjustment under random excitation (C-level and *v =* 10m/s). (a) *h*_*b*_ = 0 to 25mm (b) *h*_*b*_ = 0 to 50mm.

As mentioned in the above sections, the driving speed is one of the most important indexes for the proposed DHPS system. The fundamental dynamic properties of the proposed motor-driving DHPS subjected to different driving speeds (*v*_*b*_) have been investigated in Fig 9. Here, it will be evaluated in the quarter-car model with different *v*_*b*_, as illustrated in Figs 11 and 12. Fig 11 illustrates the response of sprung mass under the static condition from the initial position to the target height subjected to different *v*_*b*_ (40mm/s to 200mm/s).

It can be observed from Fig 11 that (1) the faster *v*_*b*_, the faster the sprung mass obtains the target position (95% target value). For example, it takes 0.40s and 0.50s (200mm/s driving speed), and 0.76s and 1.36s (40mm/s driving speed) under 25 and 50 mm required movement of *h*_*b*_, respectively; (2) the faster of *v*_*b*_, the higher overshooting of the sprung mass obtaining the target position (95% target value). For example, it reaches 19.02% (200mm/s driving speed) and 8.56% (40mm/s driving speed) under *h*_*b*_ from 0 to 25mm, and it reaches 12.96% (200mm/s driving speed) and almost no overshoot for 40mm/s under *h*_*b*_ from 0 to 50mm.

Fig 12 illustrates the response of sprung mass under typical working conditions (C-level and *v* = 10m/s) subjected to different *v*_*b*_ (40mm/s to 200mm/s).

The same phenomenon can be observed in Fig 12 as those shown in Fig 11. Here, it should be noted that the system response presented in Fig 12 is based on the open-loop strategy, which is utilized to evaluate the fundamental response properties of the proposed system. It should be noted that it is possible to obtain better response performance through an advanced control strategy, for example, taking the motor acceleration and deceleration curve into account to avoid overshooting, which will be one of the research points in the future.

**Fig 13:**
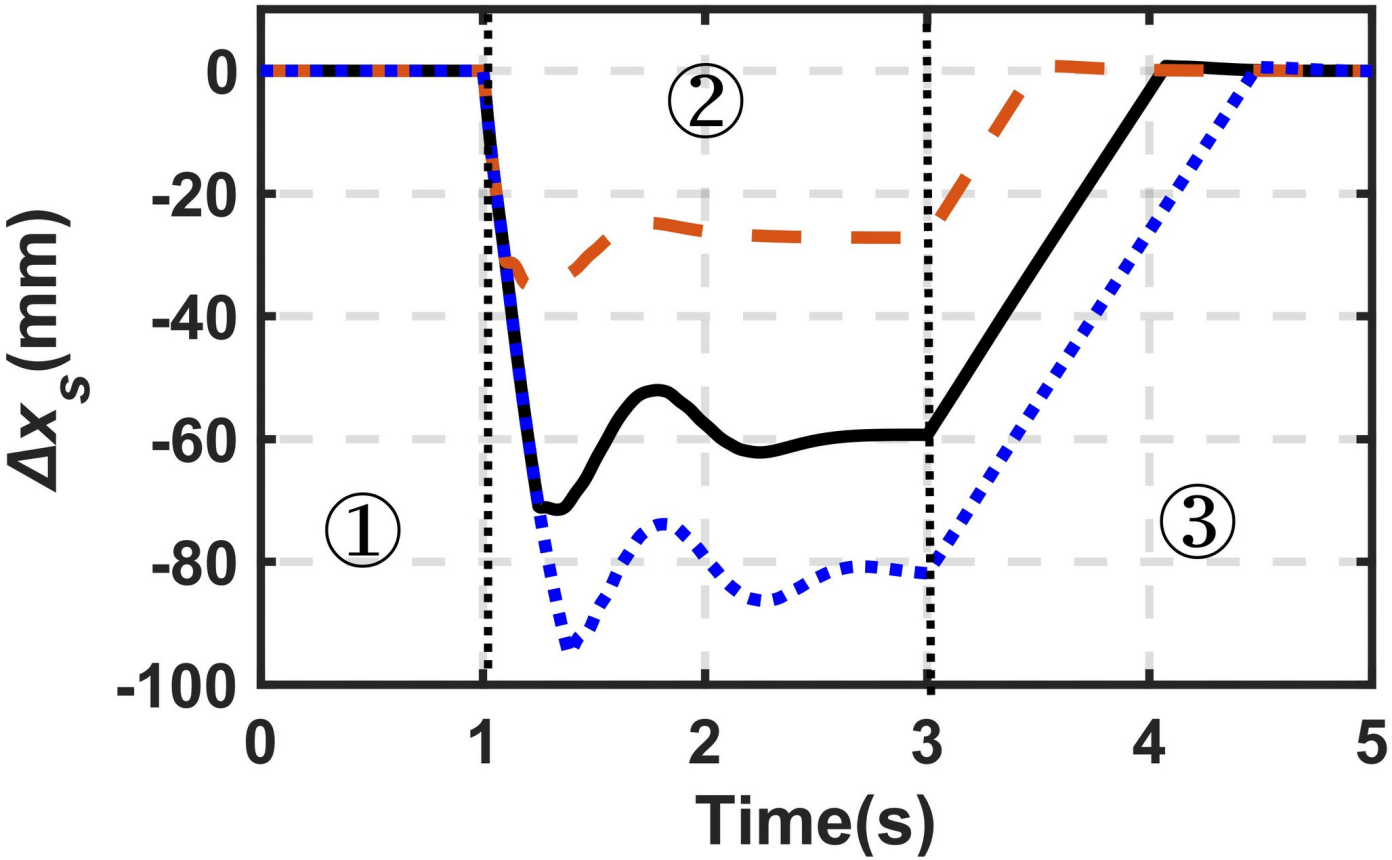
Height adjustment under different sprung masses. Dotted line (blue)-474.6kg; Solid line (black)-420kg; Dashed line (brown)-360kg.

*(2) Height adjustment subjected to different sprung masses*. Based on Eqs (2) and (7)–(9), the required value of *h*_*b*_ subjected to the command height of sprung mass (*x*_*target*_) can be evaluated as:

$$h_{b}=\frac{1}{A_{AGC}}\left[\begin{array}{c} \left(1-\sqrt[{n}]{\frac{P_{SGC0}+P_{a}}{(m_{s}+m_{l})\cdot g/A_{2}+P_{a}}}\right)V_{SGC0}-A_{2}x_{target}-\left(1-\sqrt[{n}]{\frac{P_{GC0}+P_{a}}{m_{s}g/A_{2}+P_{a}}}\right)V_{GC0^{-}}\\ \left(1-\sqrt[{n}]{\frac{P_{SGC0}+P_{a}}{\left(m_{s}g\right)/A_{2}+P_{a}}}\right)V_{SGC0}+h_{b0}A_{AGC}+\left(1-\sqrt[{n}]{\frac{P_{GC0}+P_{a}}{(m_{s}+m_{l})\cdot g/A_{2}+P_{a}}}\right)V_{GC0} \end{array}\right]$$ (18)

where *m*_*l*_ represents the mass of increased load; *h*_*b*0_ represents the initial value of *h*_*b*_. Parts ① and ② in Fig 13 illustrate the initial value of *x*_*s*_ subjected to different sprung masses, which can be evaluated from Eqs (15) and (16).

Let us set the target height (*x*_*target*_) to keep the height of the sprung mass (*x*_*s*_) under different payloads. And then the required *h*_*b*_ can be evaluated based on Eq (18). Part ③ in Fig 13 illustrates the response of sprung mass under the determined values of *h*_*b*_ for different payloads (*v*_*b*_ = 70mm/s). As expected, the *h*_*b*_ evaluated based on Eq (18) can guarantee the *x*_*target*_. Based on the designed prototype, in which the maximum length of the *h*_*b*_ is 104mm, it can be achieved under the sprung mass from 320.0kg to 474.6kg.

## 5. Conclusion

A kind of double-gas-chamber hydro-pneumatic strut (DHPS) with a continuous height adjustment function associated with constant and/or predicted stiffnesses is proposed in this paper, which is achieved by volume variation of the gas chamber through the motor-driving piston. The mathematical model is fundamentally established considering the gas models with the interacting effect of elastic force generated by two gas chambers, oil damping force model, and friction force model. The model types and model parameters are defined through experimental study applying to the designed prototype. The accuracy of the modeling is verified by experimental data under different heights and sprung masses, in which the coefficient of determination (*R*^*2*^) are all above 0.92. The performance under different excitations has been investigated based on a quarter-car model. The results show that:

1. As no extra mass of air in/out of the system, the stiffness property will be constant and/or predicted during the height adjustment procedure, the relationship between the target height and the position of the motor-driving piston are identical, which means that the final height is only decided by the value of *h*_*b*_ and can be calculated in advance.
2. The volume of the auxiliary gas chamber is modified by the designed motor-driving piston, which has the potential to provide a fast response compared with the pumping method. It has been shown that it takes 1(2) cycles to get the stable condition at 50mm/s (25mm/s) moving speed, which is achieved through a simple step motor with 8.5N∙m torque capability.
3. The height adjustment performance has been conducted on a typical quarter-car model with payloads from 320.0kg to 474.6kg.

The present work verifies the potential application of the proposed system in the vehicle suspension area, which can be easily extended to other multi-point support structures in different fields, such as ocean platforms, engineering machinery, platform tensioner-riser coupling systems and so on. However, advanced control methodologies designed to get fast response speed with low overshoot associated with extensive base-excitation experimental studies are required for future research work to meet different practical requirements.

## Supporting information

S1 Dataset (XLSX)
